# Supplementary material for: Research on the relationship between virtual social interaction and the degree of loneliness based on algorithm matching technologies: A quantitative analysis on the SOUL APP-A virtual social software for strangers
Source: PLoS One. 2024 Dec 3;19(12):e0312522. doi: 10.1371/journal.pone.0312522 (PMC11614230; doi:10.1371/journal.pone.0312522)
Supplement: Attachment — Submitted filename: PONE-D-24-18667_reviewer.pdf [file pone.0312522.s002.pdf]

PLOS ONE

Research on the Relationship between Virtual Social Interaction and the Degree of Loneliness Based on Algorithm Matching Technologies: A Quantitative Analysis on the SOUL APP-A Virtual Social Software for Strangers

--Manuscript Draft--

**Manuscript Number:**	PONE-D-24-18667
**Article Type:**	Research Article
**Full Title:**	Research on the Relationship between Virtual Social Interaction and the Degree of Loneliness Based on Algorithm Matching Technologies: A Quantitative Analysis on the SOUL APP-A Virtual Social Software for Strangers
**Short Title:**	Virtual Social Interaction and the Degree of Loneliness Based on Algorithm Matching Technologies
**Corresponding Author:**	Linsha Liu<br>Chengdu Sport University<br>Chengdu, CHINA
**Keywords:**	algorithm matching; virtual social interaction; loneliness, social anxiety; virtual social platforms
**Abstract:**	This study examines the relationship between virtual social interaction and people's social behaviors and psychology using algorithm matching technologies and questionnaire surveys. The focus is on interpersonal communication on virtual social platforms. The findings indicate that engaging in virtual social networking is often accompanied by a high level of loneliness. Users who experience social anxiety in the real world tend to feel more lonely, and this loneliness is exacerbated by presenting an unreal version of oneself and having distrust in virtual social networking. Users with higher anxiety and loneliness levels are more likely to use the algorithm matching function of virtual social networking, engage in false self-presentation, and have less trust in the platform. Since the inherent flaws of virtual social networking cannot be eliminated solely through algorithm matching, a potential solution is to introduce more offline to online social functions for strangers. This exploration of actual matching on social platforms may help reduce users' loneliness.
**Order of Authors:**	Linsha Liu
**Additional Information:**	
**Question**	**Response**
**Financial Disclosure**<br><br>Enter a financial disclosure statement that describes the sources of funding for the work included in this submission. Review the submission guidelines for detailed requirements. View published research articles from *PLOS ONE* for specific examples.<br><br>This statement is required for submission and **will appear in the published article** if the submission is accepted. Please make sure it is accurate.	The author(s) received no specific funding for this work.

Powered by Editorial Manager® and ProduXion Manager® from Aries Systems Corporation

**Funded studies**<br>Enter a statement with the following details:<br>• Initials of the authors who received each award<br>• Grant numbers awarded to each author<br>• The full name of each funder<br>• URL of each funder website<br>• Did the sponsors or funders play any role in the study design, data collection and analysis, decision to publish, or preparation of the manuscript?<br><br>Did you receive funding for this work?	
**Competing Interests**<br><br>Use the instructions below to enter a competing interest statement for this submission. On behalf of all authors, disclose any competing interests that could be perceived to bias this work—acknowledging all financial support and any other relevant financial or non-financial competing interests.<br><br>This statement is required for submission and **will appear in the published article** if the submission is accepted. Please make sure it is accurate and that any funding sources listed in your Funding Information later in the submission form are also declared in your Financial Disclosure statement.<br><br>View published research articles from *PLOS ONE* for specific examples.	The authors have declared that no competing interests exist.

Powered by Editorial Manager® and ProduXion Manager® from Aries Systems Corporation

**NO authors have competing interests**<br><br>Enter: *The authors have declared that no competing interests exist.*<br><br>**Authors with competing interests**<br><br>Enter competing interest details beginning with this statement:<br><br>*I have read the journal's policy and the authors of this manuscript have the following competing interests: [insert competing interests here]*<br><br>* typeset	
**Ethics Statement**<br><br>Enter an ethics statement for this submission. This statement is required if the study involved:<br><br>• Human participants<br>• Human specimens or tissue<br>• Vertebrate animals or cephalopods<br>• Vertebrate embryos or tissues<br>• Field research<br><br>Write "N/A" if the submission does not require an ethics statement.<br><br>General guidance is provided below. Consult the submission guidelines for detailed instructions. **Make sure that all information entered here is included in the Methods section of the manuscript.**	The research protocol underwent a thorough review and was approved by the Chengdu Sport University Research Ethics Board (2023#157)

**Format for specific study types**<br><br>**Human Subject Research (involving human participants and/or tissue)**<br>• Give the name of the institutional review board or ethics committee that approved the study<br>• Include the approval number and/or a statement indicating approval of this research<br>• Indicate the form of consent obtained (written/oral) or the reason that consent was not obtained (e.g. the data were analyzed anonymously)<br><br>**Animal Research (involving vertebrate animals, embryos or tissues)**<br>• Provide the name of the Institutional Animal Care and Use Committee (IACUC) or other relevant ethics board that reviewed the study protocol, and indicate whether they approved this research or granted a formal waiver of ethical approval<br>• Include an approval number if one was obtained<br>• If the study involved *non-human primates*, add *additional details* about animal welfare and steps taken to ameliorate suffering<br>• If anesthesia, euthanasia, or any kind of animal sacrifice is part of the study, include briefly which substances and/or methods were applied<br><br>**Field Research**<br><br>Include the following details if this study involves the collection of plant, animal, or other materials from a natural setting:<br>• Field permit number<br>• Name of the institution or relevant body that granted permission	
**Data Availability**<br><br>Authors are required to make all data underlying the findings described fully available, without restriction, and from the time of publication. PLOS allows rare exceptions to address legal and ethical concerns. See the PLOS Data Policy and FAQ for detailed information.	Yes - all data are fully available without restriction

A Data Availability Statement describing where the data can be found is required at submission. Your answers to this question constitute the Data Availability Statement and **will be published in the article**, if accepted.<br><br>**Important:** Stating 'data available on request from the author' is not sufficient. If your data are only available upon request, select 'No' for the first question and explain your exceptional situation in the text box.<br><br>Do the authors confirm that all data underlying the findings described in their manuscript are fully available without restriction?	
**Describe where the data may be found in full sentences. If you are copying our sample text, replace any instances of XXX with the appropriate details.**<br><br>• If the data are **held or will be held in a public repository**, include URLs, accession numbers or DOIs. If this information will only be available after acceptance, indicate this by ticking the box below. For example: *All XXX files are available from the XXX database (accession number(s) XXX, XXX.).*<br>• If the data are all contained **within the manuscript and/or Supporting Information files**, enter the following: *All relevant data are within the manuscript and its Supporting Information files.*<br>• If neither of these applies but you are able to provide **details of access elsewhere**, with or without limitations, please do so. For example:<br><br>*Data cannot be shared publicly because of [XXX]. Data are available from the XXX Institutional Data Access / Ethics Committee (contact via XXX) for researchers who meet the criteria for access to confidential data.*<br><br>*The data underlying the results presented in the study are available from (include the name of the third party*	All relevant data are within the manuscript and its Supporting Information files.

and contact information or URL).<br>• This text is appropriate if the data are owned by a third party and authors do not have permission to share the data.<br><br>* typeset	
Additional data availability information:	

Powered by Editorial Manager® and ProduXion Manager® from Aries Systems Corporation

手稿 Click here to access/download;Manuscript;Manuscriptwith Author Details 0515.docx

Title Page

Type of the study: Research Article

Title:

Research on the Relationship between Virtual Social Interaction and the Degree of Loneliness Based on Algorithm Matching Technologies: A Quantitative Analysis on the SOUL APP-A Virtual Social Software for Strangers

Short Title: Virtual Social Interaction and the Degree of Loneliness Based on Algorithm Matching Technologies

Linsha Liu[1]

[1] Linsha Liu, School of Journalism and Communication, Chengdu Sport University.

Corresponding author:

Linsha Liu, School of Journalism and Communication, Chengdu Sport University, 2 Tiyuan Road, Chengdu, Sichuan, P.R. China.

Zip Code 610041.

Email:liulinsha@swjtu.edu.cn.

Tel:+8613678189496.

Declarations: This study was approved by the Ethics Committee of Chengdu Sport University, with ethics approval reference 2023 [157].

Conflict of interest disclosure: I have no conflicts of interest to disclose.

Research on the Relationship between Virtual Social Interaction and the Degree of Loneliness Based on Algorithm Matching Technologies: A Quantitative Analysis on the SOUL APP-A Virtual Social Software for Strangers

Abstract. This study examines the relationship between virtual social interaction and people's social behaviors and psychology using algorithm matching technologies and questionnaire surveys. The focus is on interpersonal communication on virtual social platforms. The findings indicate that engaging in virtual social networking is often accompanied by a high level of loneliness. Users who experience social anxiety in the real world tend to feel more lonely, and this loneliness is exacerbated by presenting an unreal version of oneself and having distrust in virtual social networking. Users with higher anxiety and loneliness levels are more likely to use the algorithm matching function of virtual social networking, engage in false self-presentation, and have less trust in the platform. Since the inherent flaws of virtual social networking cannot be eliminated solely through algorithm matching, a potential solution is to introduce more offline to online social functions for strangers. This exploration of actual matching on social platforms may help reduce users' loneliness.

Key Words: algorithm matching ; virtual social interaction, loneliness ; social anxiety ; virtual social platforms

In recent years, the rapid advancement of media technologies has ushered in a new era of digital and smart media. This has led to the intelligent matching of our mental and psychological behaviors, such as thinking, actions, and cultural preferences, with the utilization of big data and smart technologies. As a result, users are now able to immerse themselves extensively in social networking experiences in a more direct manner. According to data from QuestMobile, the number of mobile networking users in China reached 1.224 billion in 2023. Since 2018, several new social networking apps have been launched, including popular apps like Zhiya, Soul, and Yinyu. These apps strive to create enriched and diversified social activities while improving platform matching efficiency and user experience through the application of algorithms, big data, and AI (Artificial Intelligence).

This study investigates the emotions of users on SOUL , a social networking app for strangers, from a theoretical perspective of Media Ecology. The aim is to examine the interconnections between users, algorithm-driven virtual social interactions, and the level of loneliness. The study aims to explore the dynamic interactions between human beings, media technologies, and social

contexts, and to uncover the potential for coexistence between media and human beings through collaborative efforts of media technologies and human practices.

Part One Research Background

Paul Levinson (1988) believes that the problems and disadvantages of media development can be addressed through the sensible selection of human beings. Additionally, media technologies should be updated to meet the actual demands of human beings. On the other hand, Prof. Shirley Turker (2014) raises the question of why the more we expect from technological benefits, the further we drift apart. She questions the influence of technologies on the real world of human beings. In the context of technological information, human beings are interconnected with others from all over the world, but they may be indifferent to those nearby. As technological development progresses, humans become weaker and more obstreperous online, while feeling lonelier in real-world society. This phenomenon has garnered significant public attention, and the phrase 'Alone Together' has become a representation of the psychological condition of netizens. In the current digital information era, we are pondering the disparities between the concepts presented in 'Alone Together' . We question whether, as technologies continue to advance, they truly align with the actual needs of human beings or if they further alienate them.

This study is based on the theoretical framework of Media Ecology and aims to investigate the dynamic influences of the co-existence of media technologies and humans. It also explores the potential of a synthetic symbolic context that emerges through their mutual interactions. The study focuses on studying the communicative and ecological systems shaped by media and the media context. The goal is to uncover the mutual interactions between media technologies, humans, and social context, and to explore possible approaches for achieving a more balanced communicative context system.

Part Two Literature Reviews & Hypotheses

I. Realistic Dilemma: Research on the Relationship between Real-world Social Anxiety of Virtual Social Users and the Degree of Loneliness

According to William Barrett (1992), human beings nowadays tend to prefer engaging with the virtual world rather than experiencing the tangible reality outside their windows. This preference has become more evident with the advancement of virtual technologies. As the virtual world becomes more realistic, individuals who are addicted to virtual social networking find it increasingly challenging to willingly engage with the real world. This raises the question: how does the user's virtual behavior impact their actual situation? Specifically, what are the relationships between the

user's virtual social behavior, degree of loneliness, and social anxiety?

Social anxiety, a common problem in interpersonal social interactions (ZHANG Yongxin, et al., 2017), is characterized by emotional experiences such as fear, tension, and anxiety during communication (LI Lingyu, 2010). Individuals with social anxiety fear interacting with strangers and worry about how they are perceived, often feeling embarrassed and ashamed of their fear and nervousness in social situations (American Psychiatric Association, 2013). Loneliness, on the other hand, refers to a subjective feeling of social isolation and the pain of rejection due to a lack of social interactions (Michela, J. L., et al., 1982). Numerous studies have shown a strong positive correlation between loneliness and social anxiety in both youth and adulthood (Spindler, H, et al., 2007). Moreover, there is statistical significance indicating that social anxiety positively predicts loneliness (SUN Mengyuan and LIU Kun, 2018). To test whether the above conclusions still hold true in the virtual social environment, we make the first hypothesis:

H1: The actual social anxiety of virtual social users positively predicts their degree of loneliness.

II. Internet Disadvantages: Studies on the Relationship between the Real-world Social Anxiety, Degree of Loneliness and Virtual Social Behavior

The relationship between the utilization of social networking and the level of loneliness experienced by users has not been clearly established in current studies, leading to different perspectives. For example, Burke, Marlow, and Lento (2010) argue that the use of social networking is positively correlated with loneliness, while Teppers, Luyckx, Klimstra, and Goossens (2014) hold the view that frequent utilization of social networking reduces loneliness. In this regard, YAO Qi, MA Huawei, YAN Huan, and CHEN Qi (2014) suggest that future research should focus on specific behaviors within social networking. Existing studies on virtual social behavior often examine the use of virtual self-presentation and virtual social trust as two types of social networking.

Self-presentation, also known as self-expression and impression management, was introduced by Goffman Erving (1959). It refers to the effort individuals make to shape the way others perceive them and to create a desired self-image. The more clear and authentic individuals are in their self-presentation, the less social anxiety they experience in face-to-face interactions (Van Dijk et al., 2014). Therefore, it can be concluded that authentic self-presentation is negatively associated with social anxiety. However, there is a gap in the literature regarding the relationship between self-presentation of virtual social networking users and their loneliness and social anxiety when considering algorithm matching technologies. Thus, this study proposes a second hypothesis to explore this relationship.

H2: The real self-presentation of virtual social users is negatively predicted by their real-world social anxiety and negatively predicted the degree of loneliness.

are related to real-world social anxiety.

H2.1: The real-world social anxiety of virtual social users negatively predicts the self-presentation of virtual social networking.

H2.2: The self-presentation of virtual social networking negatively predicts the degree of loneliness.

Previous studies have shown that social networking users with higher levels of social trust are more likely to engage in online communication and seek social interactions (Dwyer, C., et al., 2007; Sledgianowski, D. and Kulviwat, S., 2009). Specifically, in social media apps, higher levels of social trust are associated with increased time spent on online social activities (Huisman, A., et al., 2001). Users with greater social trust are also more active and open to accepting new things, indicating the mediating role of social trust in online social networking (Chen Y., et al., 2015). This study aims to investigate the mediating role of virtual social trust in the relationship between social anxiety and the degree of loneliness. As a result, the third hypothesis is formulated as follows:

H3: The social trust of virtual social networking users is negatively predicted by their real-world social anxiety, and negatively predicted the degree of loneliness.

H3.1: The real-world social anxiety of virtual social networking users negatively predicts their virtual social trust.

H3.2: The virtual social trust of virtual social networking users negatively predicts their degree of loneliness.

This study constructs a model based on the aforementioned assumptions, as shown in Diagram 1:

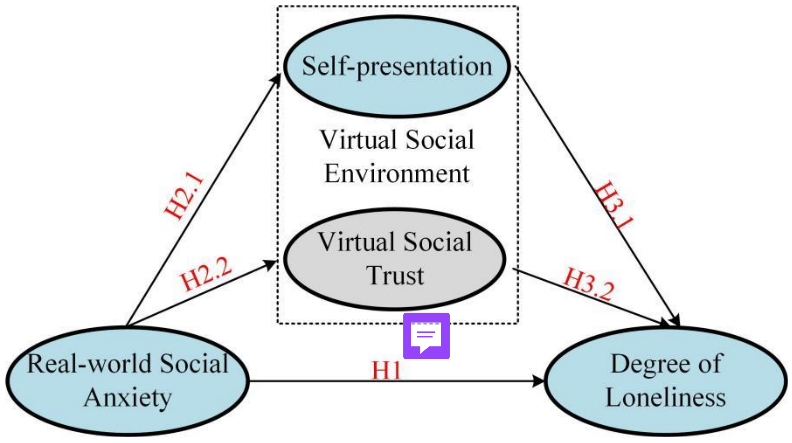

Diagram 1: Model Diagram for Virtual Social Behaviors, Degree of Loneliness, and Social Anxiety

If the above assumptions were confirmed, what are the impacts of algorithm matching technologies on the trust/self-presentation, loneliness, and real-world social anxiety of virtual social networking users?

III. The Temptation of Quick Matching: Study on The Impacts of Virtual Social Acceptance with Application of Algorithm Matching Technologies

The combination of powerful computing, large amounts of data, and precise algorithms has facilitated the widespread use of algorithm matching technologies in social media platforms (HU Shuguang and CHEN Changfeng, 2019). By effectively utilizing AI technologies and targeted algorithms, personalized recommendations and improved efficiency in matching relevant user information can be achieved. The design principle behind these algorithm matching technologies is to create social interactions that better align with the users' actual needs and reduce loneliness. Hence, the fourth hypothesis is proposed as follows:

H4: Virtual social networking users keen on algorithm matching technologies have a lower sense of loneliness

III. Looking for Satisfied Partners: Study on the Relationship of Algorithm Matching Technologies-driven Society and Virtual Social Interactions

ZHAO Jing and other scholars (2013) defined online social trust as the anticipation of conversations and commitments, both written and oral, by counterparts in interactive online social contacts involving risks. Virtual social trust is manifested by being confidential with others' information during virtual interpersonal interactions while ensuring the authenticity of one's personal information. In essence, virtual social trust implies a virtual method for mutual identity in an online context. To investigate whether the accurate matching of algorithm technologies amplifies mutual identity and virtual social trust, the fifth hypothesis is as follows:

H5: Users keen on algorithm matching technologies have a better experience in virtual social interactions.

H5.1: Users keen on algorithm matching technologies are more willing to show self-presentation in virtual social interactions;

H5.2: Users ken on algorithm matching technologies have a higher sense of trust in virtual social interactions.

Individuals who struggle with socializing in real-world society may find online social activities to be time-consuming and draining. Wang Yiling (2011) discusses this inefficiency. Fan Ziqing and Gao Yan (2016) analyze the emotional and psychological effects of online social activities on real-world social interactions. Yang Siqing (2016) explores the challenges faced by college students in the digital age and highlights how virtual social activities can reduce their interpersonal skills in the real world. Furthermore, with the rise of algorithm matching technologies in virtual social activities,

individuals who have become accustomed to this process may encounter difficulties in engaging in real-world social interactions. This leads to the formulation of the sixth hypothesis:

H6: Users keen on algorithm matching technologies show more social anxiety in the real world.

Based on previous research, this study aims to summarize the key factors that influence the level of loneliness in real-world social activities. Additionally, it explores the impact of algorithm matching technologies on the degree of loneliness in virtual social interactions, considering factors such as factual self-presentation, interpersonal trust, and real-world social anxiety.

Part Three Design and Methodologies of Research

I. Selection of Samples

SOUL APP is a social networking platform application that emerged in 2015, aiming to alleviate users' sense of loneliness by introducing social partners who share many commonalities through data matching technologies. Additionally, data from 2023 revealed that SOUL App has a large number of active users on a daily basis and has the highest average release rate and Z generation penetration rate among similar software in China(Just So Soul, 2023). Therefore, taking SOUL users as research objects has a good representation and can fully reveal the problems involved in this study.

Data collection for this study from Nov. 1, 2023 to Nov[illegible], 2021. Among the 432 online collected questionnaires, 368 were deemed effective, resulting in an effective recovery ratio of 85.2%. For data analysis, SPSS 25.0 was used for descriptive analysis of the samples, while AMOS 25.0 was used for confirmatory factor analysis and hypothesis testing. The reliability test conducted revealed a Cronbach's Alpha coefficient of 0.768.

Ethical considerations were of utmost importance in this study. The study strictly adhered to ethical guidelines, which included obtaining informed consent, ensuring participant confidentiality, and protecting the rights and well-being of the participants. This study used anonymous online questionnaires to collect data and did not collect any personally identifiable information that could identify participants. At the beginning of filling out the online questionnaire, all potential participants were clearly informed of the purpose, methodology, potential risks and benefits of the study, and their voluntary participation was obtained. The questionnaire clearly informs participants that by submitting the questionnaire online, they are considered to have consented to participate in the study. The research protocol underwent a thorough review and was approved by the Chengdu Sport University Research Ethics Board (2023#157)

II. Variables Measurement

i. Anxiety on real-world social activities

Referring to the Interpersonal Relationship Integrative Diagnostic Scale (IRIDS) developed by ZHENG R(1996) to measure real-world social abilities, this article evaluates the current situations of the participants' real-world social abilities, specifically focusing on their conversations and categories of friendship. The revised version of the scale includes five questions and use a five-point scoring system, with 1-point indicating a lack of match and 5-point indicating full compliance. The Internal Consistency Reliability value in this study is 0.898, and the KMO and Bartlett's Test of Sphericity values are 0.827 ($p<0.001$).

ii. The Scale of the degree of loneliness

Referring to the simplified version of the Scale of the degree of Loneliness (Hughes, M. E, et al., 2004) and the mental health assessment scale by WANG X, et al. (1999), this study establishes four questions to assess the degree of loneliness experienced by the participants. The degree of loneliness is measured on a five-grade scoring system, with a higher score indicating a higher degree of loneliness. The Internal Consistency Reliability value in this study is 0.832, and the KMO and Bartlett's Test of Sphericity values are 0.693 ($p<0.001$).

iii. Real Self-presentation on virtual social

Learning from the positive self-presentation and questionnaires developed by Kim, J., and Lee, J.E. (2011), and co-translated by NIU G. and other scholars (2015), this study applies a six-question scale to measure the extent of factual self-presentation on virtual social platforms. A five-grade scoring system is employed, where 1-point indicates no match and 5-point indicates full compliance. The scale demonstrates good reliability with an Internal Consistency Reliability value of 0.907, and the KMO and Bartlett's Test of Sphericity values are 0.887 ($p<0.001$).

iv. Virtual social interpersonal trust

The Online Interpersonal Trust Scale developed by DING Daoqun (2005) and the Online Interpersonal Trust Scale created by NIU Jing and MENG Xiaoxiao (2019) were utilized to assess virtual social interpersonal trust. This section consists of five questions to measure online interpersonal trust in the SOUL APP. And it is scored on a five-point scale, where 1 indicates no trust and 5 indicates complete trust. A higher score indicates a higher level of virtual trust, and vice versa. The Internal Consistency Reliability alpha coefficient for this scale was found to be 0.908, and the KMO and Bartlett's Test of Sphericity yielded values of 0.803 ($p < 0.001$).

v. **Effects of algorithm matching-driven technologies**

This study aims to explore the relationship between environment and service by using the ACSI (American Customer Satisfaction Index Model) and SSI (Student Satisfaction Inventory) (BAI Changhong and LIAO Wei, 2001). This non-scale part mainly touches up[illegible]he aspects of the effects of algorithm matching and the preferences on algorithm matching technologies with seven questions. This section is scheduled to present questions rather than scale structures.

Part Four Results

I. Information for Reference

In this study, the proportion of male participants is 48.9%, while female participants make up 51.1%. The post-95s accounts for 75%. The features of the collected samples through the questionnaires are generally consistent with those of SOUL APP users showing in the SOUL Hong Kong stock market prospectus in 2023.

II. Study on the Relations of the Variables

The relationships between real-world social anxiety, virtual social trust, self-presentation, and the degree of loneliness are presented in Table 1. Based on the statistics, the mean values for real-world social anxiety, virtual social trust, and self-presentation, as well as the degree of loneliness, exceed 3. The results indicate a positive correlation between virtual social trust and self-presentation ($r=0.658$, $p<0.001$), as well as between the degree of loneliness and real-world social anxiety ($r=0.520$, $p<0.001$). Conversely, real-world social anxiety is negatively associated with both virtual social trust and self-presentation. Specifically, real-world social anxiety is inversely related to virtual social trust ($r=-0.636$, $p<0.001$), and it is also negatively associated with self-presentation ($r=-0.766$, $p<0.001$). Similarly, the degree of loneliness is negatively associated with both virtual social trust and self-presentation. The degree of loneliness is inversely related to virtual social trust ($r=-0.471$, $p<0.001$), and it is also negatively associated with self-presentation ($r=-0.587$, $p<0.01$).

Table 1：The Correlation Coefficient Matrix, Means, Standard Deviation, Correlation Coefficient, and Square root of AVE（N=368）

Item	Real-world social anxiety	Self-presentation	Virtual social trust	Loneliness
Real-world Social Anxiety	0.807			
Self-presentation	-0.766***	0.799		
Virtual Social Trust	-0.636***	0.658***	0.823	
Loneliness	0.520***	-0.587**	-0.471***	0.762

Mean	3.122	2.810	2.667	3.196
SD	0.997	0.857	0.906	0.685

Notes：*p<0.05, **p<0.01, ***p<0.001; the value on diagonal represents the square root of AVE

III. Analysis of Confirmatory Factors

The figures in Table 2 indicate that the variables in the corresponding questions have a capacity exceeding 0.5 and fluctuate below $p<0.001$. Additionally, the Average Variance Extracted (AVE) of the variable exceeds 0.5, and the square root of AVE is greater than the correlation coefficient (as shown in Table 2). This suggests that the variables have good discriminatory validity (Fornell, C., and Larcker, D. F.,1981). Furthermore, the Composite Reliability (CR) value exceeding 0.8 indicates positive reliability of the variables in this study (Kline,R. B.,1998).

Table 2: Analysis of Confirmatory Factors（N=368）

Latent Variable	Item	Parameter Estimate					Convergence Validity		
		NSF	S.E.	t	p	SF	SMC	CR	AVE
Real-world Social Anxiety	T1	1				0.757	0.573	0.903	0.651
	T2	1.076	0.123	8.768	***	0.775	0.601		
	T3	1.011	0.12	8.419	***	0.845	0.714		
	T4	0.918	0.121	7.618	***	0.876	0.767		
	T5	0.971	0.128	7.598	***	0.773	0.598		
Self-presentation	T6	1				0.935	0.874	0.912	0.639
	T7	1.878	0.372	5.054	***	0.832	0.692		
	T8	1.721	0.351	4.911	***	0.86	0.74		
	T9	1.972	0.382	5.156	***	0.771	0.594		
	T10	1.779	0.35	5.084	***	0.821	0.674		
	T11	2.112	0.397	5.323	***	0.508	0.258		
Virtual Social Trust	T12	1				0.824	0.679	0.912	0.677
	T13	0.755	0.099	7.663	***	0.72	0.518		
	T14	0.884	0.098	9.007	***	0.808	0.653		
	T15	0.923	0.093	9.95	***	0.865	0.748		
	T16	1.015	0.098	10.313	***	0.886	0.785		
Loneliness	T17	1				0.694	0.482	0.84	0.581
	T18	1.27	0.166	7.656	***	0.897	0.805		

	T19	1.249	0.163	7.651	***	0.896		0.803	
	T20	0.604	0.14	4.327	***	0.482		0.232	
Notes: *p<0.05, **p<0.01, ***p<0.001.									

IV. Tests of Hypotheses

To investigate the relationship between real-world social anxiety, degree of loneliness, and virtual social trust and self-presentation, this study utilized AMOS25.0 to conduct SEM (Structure Equation Modelling). The aim was to examine the interrelated influences and analyze the direct effect, total effect, and mediation effect. The initial results were then revised and updated using Modification Indices. The fit of the model was assessed using Chi-square values, with a significance probability of $p=0.079>0.05$, indicating a good fit. If the hypothesis is confirmed, it suggests that the observed figures align closely with the supposed modelling. Table 3 presents the findings, showing that the ratio of CMIN and DF of SEM is lower than 3, and the NFI, TLI, and IFI exceed 0.9, indicating a good fit. The RMSEA below 0.08 also demonstrates a good fit for SEM. Additionally, the CFI, GFI and AGFI exceed 0.7, indicating an acceptable extent of SEM fit.

Table 3 Results of Common Fit Indexes for SEM (N=368)

Index	CMIN/DF	GFI	AGFI	CFI	RMESA	NFI	TLI	IFI
Value	1.177	0.869	0.795	0.984	0.044	0.903	0.977	0.984

i. Tests of Direct Effect, Total Effect, and Mediation Effect

Based on the data in Diagram 3, it can be observed that real-world social anxiety has a positive impact on users' level of loneliness ($\beta=0.341$, $p<0.001$). This means that individuals with higher levels of real-world social anxiety experience a greater degree of loneliness. Therefore, the first hypothesis (H1) is supported by relevant evidence. Additionally, real-world social anxiety has a negative effect on self-presentation ($\beta=-0.354$, $p<0.001$). This suggests that individuals with higher levels of real-world social anxiety tend to present themselves in an unrealistic manner, confirming

H2.1. Furthermore, real-world social anxiety also has a negative influence on the user's virtual social trust (β =-0.588, $p<0.001$). Users with higher levels of real-world social anxiety exhibit lower levels of trust in virtual social interactions, confirming H3.1. The self-presentation in virtual social interactions also has a negative impact on the level of loneliness (β =-0.180, $p<0.05$). This implies that the more individuals engage in untrue self-presentation in virtual social settings, the higher their level of loneliness becomes, confirming H2.2. Similarly, virtual social trust has a negative effect on the level of loneliness (β =-0.317, $p<0.001$). This indicates that individuals who have more trust in virtual social interactions tend to experience less loneliness in real society, supporting H3.2. Overall, all the proposed hypotheses have been confirmed and have revealed a paradoxical relationship between the level of loneliness and real-world social anxiety. To mitigate loneliness, it is important to engage in more authentic self-presentation and develop trust in virtual social activities. However, due to anxiety and frustrations, individuals using virtual social platforms are often hesitant to present their true selves and have limited trust in such activities.

Diagram 2: Model Diagram of Virtual Social Behavior, Real-world Loneliness, and Social Anxiety

In this study, the bias-corrected Bootstrap testing method is used to analyze the total effect and mediation effect of virtual social behavior, real-world loneliness, and social anxiety. To determine the confidence interval of the mediation effect, we estimate the value to be approximately 2.5, with the 97.5th percentile being the primary reference point. The results will be presented in Table 4.

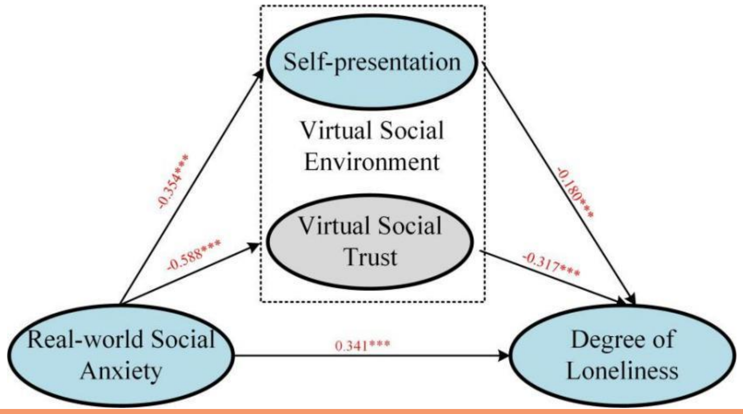

Table 4 Bootstrap Results (N=368)

Route	Effect	Estimate	Bootstrap SE	z	BC95% CI		p	Mediation Effect Ratio
					MAX	MIN		
A→（B+C）→D	Total	0.385	0.135	2.852	0.696	0.162	0.001	0.712
	Direct	0.111	0.147	0.755	0.402	-0.174	0.451	

	Indirect	0.274	0.149	1.839	0.665	0.055	0.019	

Notes: A represents real-world social anxiety, B represents real self-presentation C represents virtual social trust, and D represents the degree of loneliness.

The results indicate(Table 4) a significant positive relationship between real-world social anxiety and the degree of loneliness (p<0.05).That self-presentation and virtual social trust act as mediators between real-world social anxiety and the degree of loneliness, accounting for approximately 71.2% of the mediation effect. According to the standards for testing mediation effects (MacKinnon D.P., et al., 2002), a mediation effect is considered distinctive when the indirect effect Z exceeds 0.9 and P<0.05. However, if Z is lower than 1.96, the mediation effect is deemed incomplete. In the context of the structural equation model (SEM) analyzing virtual social behavior, real-world loneliness, and social anxiety, the observed Z value of 1.839 indicates an obvious incomplete mediation effect, suggesting the presence of other potential mediating variables.

ii. Studies on the Fourth, Fifth and Sixth Hypotheses

By conducting a mean of independent sample T-Test on matching pairs, this study examined the correlation between algorithm matching technologies and the degree of loneliness among users. The results, presented in Table 5, indicate that there is a significant difference in the extent of preferences for algorithm-matching users in terms of loneliness (p<0.001). Users who like algorithm matching have a higher degree of loneliness (M=3.35) compared to those who dislike it (M=2.964). However, the loneliness disparities between users who like algorithm matching (M=3.35) and those who are indifferent (M=3.378) are not statistically significant (p=0.753>0.05). On the other hand, there was a significant difference in loneliness scores (p<0.001) between users who dislike algorithm matching and those who are indifferent. Users who dislike algorithm matching reported lower levels of loneliness in the real world, and this group differed significantly from the other types. Therefore, the result of H4 (Hypothesis 4) is negative.

Table 5: Disparities in Loneliness of User's Preferences on Algorithm Matching

Preferences	Like			Dislike			Indifference		
	n	M	SD	n	M	SD	n	M	SD
Loneliness	120	3.350	0.785	84	2.964	0.687	164	3.378	0.679

By comparing the mean of independent sample T-Test in matching pairs, this study aims to explore the correlation between algorithm matching technologies and real self-presentation, based on the scale means of self-presentation and preferences on algorithm matching in virtual social settings. The results, as shown in Table 6, indicate that there is a significant difference in the extent

of preferences on algorithm-matching users in terms of real self-presentation (p0.05). However, there are significant differences ($p<0.001$) in self-presentation between users who dislike algorithm matching and those who are indifferent. Users who dislike algorithm matching prefer to show their real self-presentation on virtual social platforms, and their data also significantly differs from the other groups.

Table 6: Disparities in Self-presentation of User's Preferences on Algorithm Matching

Preferences	Like			Dislike			Indifference		
	n	M	SD	n	M	SD	n	M	SD
Self-presentation	120	2.667	0.927	84	3.222	0.891	164	2.703	0.713

By comparing the mean of independent sample T-Test in matching pairs, this study aims to determine the correlation between algorithm matching technologies and virtual social trust. The results, shown in Table 7, indicate that there are slight disparities in real self-presentation based on the extent of preferences on algorithm-matching users ($p=0.059>0.05$). On one hand, users who like algorithm matching have a mean real self-presentation score of 2.68, while those who dislike it have a mean score of 2.933. On the other hand, there are also slight disparities in real self-presentation ($p=0.157>0.05$) between users who like algorithm matching ($M=2.68$) and those who are indifferent ($M=2.522$). However, the disparities in self-presentation between users disliking algorithm matching and those who are indifferent are significant ($p<0.001$). Users who dislike algorithm matching tend to show more real self-presentation on virtual social platforms, and this data differs significantly from the other categories. The relationship between real self-presentation and virtual social trust is positively interrelated, as confirmed in Table 3 ($r=0.658$, $p<0.01$) and repeatedly supported by H5. In summary, H5 suggests that users who like algorithm matching do not necessarily show more authentic self-presentation or have more virtual social trust.

Table 7: Disparities in Virtual Social Trust of User's Preferences on Algorithm Matching

Preferences	Like			Dislike			Indifference		
	n	M	SD	n	M	SD	n	M	SD
Virtual Social Trust	120	2.680	0.988	84	2.933	0.859	164	2.522	0.838

By conducting a mean comparison using independent sample T-Test on matching pairs, this study aimed to explore the correlation between algorithm matching technologies and real social anxiety, based on the scale means of virtual social trust and preferences. The results, presented in Table 8, indicate significant differences in the extent of preferences among algorithm-matching users in terms of real self-presentation ($p<0.001$). Users who like algo[illegible]n matching ($M=3.387$)

exhibit higher levels of real-world social anxiety compared to those who dislike it ($M=2.819$). Additionally, significant disparities in real-world social anxiety are observed between users who like algorithm matching ($M=3.387$) and those who are indifferent ($M=3.083$) ($p=0.013<0.05$). Similarly, there are noticeable differences in real-world social anxiety between users who dislike algorithm matching and those who are indifferent ($p=0.025<0.05$). These findings suggest that users who prefer algorithm matching experience greater anxiety in real social interactions, and also support hypothesis H6.

Table 8: Disparities in Real-world Social Anxiety of User's Preferences on Algorithm Matching

Preferences	Like			Dislike			Indifference		
	n	M	SD	n	M	SD	n	M	SD
Real-world Social Anxiety	120	3.387	1.1617	84	2.819	0.910	164	3.083	0.855

Part Five Conclusions

I. Virtual Social Exacerbates the Degree of Loneliness

The results showed that virtual social interactions actually increased feelings of loneliness. Additionally, real-world social anxiety had a direct impact on loneliness to some extent. Interestingly, there were correlations between real social anxiety, virtual social trust (real self-presentation), and loneliness. Users often turned to virtual social interactions to alleviate their loneliness caused by real social activities. However, due to negative experiences in real social situations, virtual social users tended to present themselves as more unreal and have less trust, which exacerbated their loneliness. Shirley's viewpoints were confirmed and it was found that lonelier users in real society tended to be more active on virtual social platforms. It was not the virtual social activity itself that amplified feelings of loneliness, but rather the trust and attitude towards virtual social interaction. The findings demonstrated that unreal self-presentation and mistrust increased feelings of loneliness in virtual social interaction. In this context, mutual trust plays a crucial role in determining the psychological effects of virtual social interaction(JIN Xin, et al., 2017). Genuine social trust is what reduces loneliness in virtual social interactions. Research also shows that individuals with higher levels of loneliness are hesitant and fearful of self-presenting, lack trust in others, and carry real-life frustrations and self-humiliation into virtual social situations. This negative attitude towards real social situations affects virtual social interactions, leading to decreased mutual trust and increased concerns about negative comments, ultimately exacerbating

social anxiety. Consequently, these users also fear negative comments on virtual social activities and experience conflicts with the identity and amity of other virtual social users, further intensifying their feelings of loneliness.

II. Inefficient Matching: Camouflages Lead to Inevitable Loneliness

Regarding Hypotheses 4, 5, and 6, the main conclusion is that users who experience higher levels of loneliness and real social anxiety tend to engage in unreal self-presentation and exhibit lower levels of virtual social trust when utilizing the algorithm matching feature on the SOUL APP. These findings are not consistent with the intended purpose of the SOUL APP.

Individuals with higher levels of real social anxiety are not only motivated to make a positive impression on others but also care about their social skills (Schlenker, B. R., and Leary, M. R., 1982). Consequently, individuals who experience anxiety in online social settings tend to focus more on maintaining their self-image and self-improvement, which can lead to a reduced level of trust (LIN Gongcheng, et al., 2016). Moreover, individuals with higher levels of real social anxiety often have a negative perception of their own image, characteristics, and behaviors, and may lack self-identity. As a result, they tend to be pessimistic about sharing their personal information with society through online platforms. These individuals with lower self-awareness are particularly susceptible to the influence of negative online social comments (WU Yang, et al., 2020). Furthermore, individuals with more negative attitudes towards social anxiety are more prone to self-suspicion. Continuous experiences of rejection in social interactions can significantly impact their self-worth and result in low self-esteem (ZHANG Weiling, 2016). On the other hand, some users strive to create an ideal image through self-disclosure, but the information they share can be a mix of authentic and fabricated messages. Individuals with weaker social abilities in online social contexts may struggle to differentiate between reality and the performance they present online (YU Guoming, et al., 2019). Additionally, the degree of loneliness is found to be negatively associated with the self-presentation of online chatting. Users who are dissatisfied with online social experiences tend to feel more lonely (Leung, L., 2003). The inconsistent social activities that are based on a fake or camouflaged self-image indicate a level of loneliness and social anxiety. As a result, these users find it difficult to select a partner who is algorithmically matched and shares commonalities, because these partners are those whom they are self-denial about. Virtual social users aim to find a perfectly compatible partner with whom they can engage in mutual interactions.

Additionally, considering a different viewpoint, can algorithm technologies-driven matching truly satisfy the actual demands of users? In this context, the initial step of algorithm-matching-driven online social platforms involves not just communication between users, but also the more common human-computer interactions. The significance of AI lies in the specific relationship between humans and computers, rather than the technology itself or the subjective imagination of

humans (M. Coeckelbergh, 2014). AI possesses a distinct advantage in terms of objectivity and efficiency in data processing. However, when it comes to the inherently [illegible]mplex process of social interactions, which involve subjective preferences and intricate thinking, the existing algorithm matching technologies struggle to fully comprehend and accurately capture the exact preferences of social partners. Perhaps, with advancements in AI technologies, the potential lies in exploring the use of decoding technologies such as electrocorticogram to guide human-computer interactions. These advancements could potentially offer new insights into the possibilities of direct exchanges of ideas.

IV. To Address the Problems or Compromise: Possibilities of Future Social for Strangers

Regarding the aforementioned factors, virtual social users should overcome virtual social barriers and present their real selves, demonstrating trust in their partners. When SOUL APP users become tired of shaping their self-image, they can log off their SOUL account, which will then remove their list of friends. The SOUL system will then recommend new partners based on the provided information. In this way, the soul matching function acts as a matching game. It is important to note that algorithm matching technologies do not simply enable quick social matching for virtual social users, but they also struggle to capture the true essence of virtual social interactions. Communicating with strangers online requires caution and doubt, and building mutual trust is crucial for algorithm matching-driven virtual social activities. However, it is important to recognize that the emotional impact of face-to-face communication far surpasses that of virtual social interactions. Furthermore, there are also drawbacks to algorithm matching-driven virtual social activities. When virtual social users heavily rely on algorithm matching, they become accustomed to virtual standards and habits of interaction, which can complicate real social communication. Additionally, virtual social platforms can lead to the development of split personalities and a blurred self-definition, as online users have the ability to shape multiple self-images and engage with a wide range of personalities and perspectives (Valkenburg, P. M., & Peter, J., 2011). When users willingly subject themselves to technology filtering, they may find it increasingly difficult to think independently and critically. With the increasing reliance of online users on algorithm matching technologies, the fragmented mindset formed by various scattered information will dominate the thinking pattern of users, resembling the concept of a one-dimensional man as defined by Herbert Marcuse.

To address the limitations of virtual social interactions among strangers, it is advisable to create a combination of online and offline social activities that allow users to present their real selves. Additionally, providing guidance on socializing in different situations can foster closer relationships among a wider range of users. Tailored cooperation can strengthen social bonds, as trust built through collaboration supports the development of interpersonal connections. A key factor in

successfully engaging in real social interactions with strangers is proximity. Being physically close or sharing common localities makes it easier for strangers to engage in real social activities. The advancements in 5G technology, coupled with quick and accurate Navigation Positioning Systems, facilitate the development of distance-matching technologies that efficiently connect individuals. Within this technological context, it becomes feasible to create platforms that facilitate vertical socializing with strangers, allowing for more authentic scenarios. This approach effectively reduces anxiety associated with face-to-face interactions and mitigates feelings of loneliness.

Notes:
No funding was received.

References

American Psychiatric Association. (2013). Diagnostic and Statistical Manual of Mental Disorders. 5th ed. Washington, DC: American Psychiatric Association.

Bagozzi, R. P., and Yi, Y. (1988). On the Evaluation of Structural Equation Models. Journal of the Academy ofMarketing Science,16(1):74-94.

BAI Changhong and LIAO Wei. (2001). Analysis on Customers’ Satisfaction Based on Customer Perception . Journal of Nankai University (Philosophy and Social Science Edition). 6, 14-20.

Bollen, Kenneth A., and Long, J. Scott.(1993).Testing Structural Equation Models. Sage Focus Editions. Newbury Park: Sage Publications.

Byrne, B. M. , and Erlbaums, L..(2009). Structural equation modeling with AMOS: basic concepts, applications, and programming. Routledge.

Carmines E G, and McIver J P.(1981). Analysing Models with Unobservable Variables. Bohrnstedt G W, Borgatta E F. Social Measurement Current Issues. Beverly Hills, Calif: Sage Publications, 65-115.

CHEN Y., Yan X., Fan W., and Gordon M. (2015). The Joint Moderating Role of Trust Propensity and Gender on Consumers’ Online Shopping Behavior. Comput. Hum. Behav. 43, 272–283.

DING Daoqun and SHEN Mowei.(2005). The Relationship between Online Social Support and Online Interpersonal Trust. Psychological Science.2, 30-303.

Dwyer, C., Hiltz, S.R., and Passerini, K. (2007).Trust and Privacy Concern Within Social Networking Sites. A Comparison of Facebook and MySpace. In Proceedings of the 13th Americas Conference on Information Systems, AMCIS 2007, Keystone, CO, USA, 9-12 August 2007.

FAN Ziqing and GAO Yan.(2016). Striking a Balance between Contemporary College Students' Social Networking and Social Reality . Legal Expo. 10, 66.

Fornell, C., and Larcker, D. F.(1981). Evaluating Structural Equation Models with Unobservable Variables and Measurement Error. Journal of Marketing Research. 18(1), 39–50.

Goffman, E.(1959). On Face-Work: Interactions Ritual: Essays on Face-to-Face Behavior, NY: Garden City, Anchor.5.

Hughes, M. E., Waite, L. J., Hawkley, L. C., and Cacioppo, J.A.(2004). Short Scale for Measuring Loneliness in Large Surveys Results from Two Population-based Studies. Research on Aging. 26(6), 655–672.

Huisman, A., Eijnden, R.V.D., and Garretsen, H.(2001).'Internet addiction' - A call for systematic research. JOURNAL OF SUBSTANCE USE. 6(1),7-10.

HU Shuguang, CHEN Changfeng.(2019) Concepts and Norms: Dilemma and Guidance of Media Ethics in the Era of Artificial Intelligence . China Press. 2, 11-15.

JIANG Xiaoli and JIA Ruili. (2017). Gamification: A Prominent Communication Gene-From the Perspective of Humanization Trend Theory of Media Evolution . China Editor. 8, 8-13.

JIN Xin, LI Yanmei, LI Xiashu, YANG Liqian, and LUO Yuchen.(2017). The Relationship between Online Social Attitude, Online Trust, Interpersonal Trust, Social Anxiety, and Loneliness [J]. Chinese Journal of Clinical Psychology. 1, 185-187+16.

Just So Soul.(2023). 2023 Generation Z Double Eleven consumption behavior report. https://www.163.com/dy/article/IJ689FKL0511D2LM.html.[Accessed November 10th, 2023].

Kim, J., and Lee, J. E.(2011). The Facebook Paths to Happiness: Effects of the Number of Facebook Friends and Self-presentation on Subjective Well-being. Cyberpsychology, Behavior, and Social Networking. 14(6), 359-364.

Kline, R. B.(1998) Principles and Practice of Structural Equation Modeling. New York, NY: The Guilford Press. 31.

Leung, L.(2003). Impacts of Net-generation Attributes, Seductive Properties of the Internet, and Gratifications-obtained on Internet Use.Telematics and Informatics. 20(2), 107-129.

LI Lingyu. (2010). The Relationship between Social Anxiety, Attachment and Mental Health of College Students. Chinese Journal of Health Psychology. 18(2), 186-189.

LIN Gongcheng, LI Ying, and CHEN Jinyun.(2016). Social Anxiety, Self-disclosure and Online Interaction Among College Students: An Analysis of Microblog Social Behavior. Youth Research. 4, 29-37.

MacKinnon, D.P., Lockwood, C. M., Hoffman, J. M., West, S.G., and Sheets, V. A.(2002).Comparison of Methods to Test Mediation and Other Intervening Variables effects. Psychological Methods.7(1), 83–104.

M.Coeckelbergh.(2014).The Moral Standing of Machines: Towards a Relational and Non-Cartesian Moral Hermeneutics. Philosophy and Technology. 27(1).

Michela, J.L., Peplau, L.A., and Weeks, D.G.(1982) .Perceived dimensions of attributions for loneliness. Journal of Personality & Social Psychology, 43(5), 929-36.

Moira, B., Cameron, M. and Thomas, L. (2010). Social network activity and social well-being. Paper presented at Human Factors in Computing Systems pp. 1909-1912.

NIU Gengfeng, BAO Na, ZHOU Zongkui, FAN Cuiying, KONG Fanchang, and SUN Xiaojun.(2015). The Influence of Virtual Socializing Self-presentation on Life Satisfaction: The Role of Positive Emotion and Social Support. Psychological Development and Education. 31(05), 563-570.

NIU Jing and MENG Xiaoxiao.(2019). The Effect of Social Media Trust on Privacy Risk Perception and Self-disclosure: The Mediating Effect of Online Interpersonal Trust. International Press. 7, 91-109.

Paul Levinson (1988).Mind at Large: Knowing in the Technology Age, Greenwich,CT: JAI Press,12.

QuestMobile(2023). 2023 China Mobile Internet Annual Report. [EB/OL].http://news.china.com.cn/mts/2024-01/31/content_1190299.htm.

Schlenker, B. R., and Leary, M. R.(1982). Social Anxiety and Self-presentation: A Conceptualization Model. Psychological Bulletin. 92(3), 641-669.

Shirley, T. (2014). Alone Together . Translated by ZHOU Kui and LIU Jingjing. Hangzhou: Zhejiang People's Publishing House.

Sledgianowski, D.and Kulviwat, S.(2009). Using Social Network Sites: The Effects of Playfulness, Critical Mass and Trust in A Hedonic Context. J. Comput. Inf. Syst. 49, 74–83.

Spindler, H., Pedersen, S.S., and Serruys, P.W.(2007).Type-D Personality Predicts Chronic Anxiety Following Percutaneous Cronary Intervention in the Drug-eluting Stent Era. J Affect Disord, 99(1-3), 173-179.

SUN Mengyuan and LIU Kun. (2018). Analysis of the Mediating Effect of Loneliness between Social Anxiety and Depressive Symptoms in Middle- school Students. China Health Statistics, 35(06):926-928.

Teppers, E., Luyckx, K., A. Klimstra, T.,and Goossens, L. (2014). Loneliness and Facebook Motives in Adolescence: A Longitudinal inquiry into Directionality of Effect. Journal of Adolescence, 37(5), 691-699.

Valkenburg, P. M., and Peter, J.(2011). Online Communication among Adolescents: An Integrated Model of Its Attraction, Opportunities, and Risks. Journal of Adolescent Health. 48(2), 121-127.

Van, Dijk, M. P., Branje, S., Keijsers, L., Hawk, S. T., Hale III, W. W., and Meeus, W. (2014). Self-Concept Clarity across Adolescence: Longitudinal Associations with Open Communication with Parents and Internalizing Symptoms. Journal of Youth and Adolescence, 43, 1861-1876.

WANG Xiangdong, WANG Xilin, and MA Hong.(1999). Handbook of Mental Health Rating Scale. Beijing: Chinese Journal of Mental Health.101.

WANG Yiling.(2011). Network Interpersonal Communication and the Sense of Belonging in Network Community: An Empirical Study on The Netizen in Coastal Cities. Journal of Journalism University. 1, 82-92.

William, B. (1992). Irrational Man-Studies on Existentialism. Shanghai: Shanghai People's Publishing House. 286.

WU Yang, WU Li, NIU Gengfeng, CHEN Zhenzhen, and LIU Lizhong. (2020).The Influence of WeChat Moments on College Students’ Depression: The Negative Role of Social Comparison and Self-concept Clarity in Depression among College Students . Psychological Development and Education. 4, 86-493.

YANG Siqing. (2016).Factual Social Barriers of College Students in the Internet Era and To Overcome These Barriers. Science & Technology Innovation and Productivity. 8, 63-65+68.

YAO Qi, MA Huawei, YAN Huan, and CHEN Qi. (2014). Analysis of Individual Behavior of Social Network Users from the Perspective of Psychology. Advances in Psychological Science, 22(10), 1647-1659.

YU Guoming, ZHU Yangshu, ZHANG Manqi, and WANG Zhian.(2019). Research on Weak Relationships in Network Communication: Control Mode and Path Efficiency: A Case Study of Stranger Social APP. Journal of Southwest University for Nationalities (Humanities and Social Science Edition). 9, 141-146.

ZHANG Weiling.(2016). The Mediating Role of Self-esteem in Self-acceptance and Social Anxiety among College Students . Chinese School Health. 9,1354-1357.

ZHANG Yongxin, ZHOU Zongkui, and ZHU Xiaowei, and LIAN Shuailei. (2017). The Influence of Social Networking on Adolescents' Well-being: A Moderated Mediation Model. Psychological and Behavioral Research. 15 (02), 189-196.

ZHAO Jing, SUN Xiaojun, ZHOU Zongkui, WEI Hua, and NIU Gengfeng.(2013). Interpersonal Trust in Online Communication. Progress in Psychological Science. 8, 1493-1501.

ZHENG Richang.(1996). Psychological Diagnosis of College Students. Jinan: Shandong Education Press. 78.
